# High burden of CMV infections after simultaneous pancreas-kidney transplantation—a nationwide cohort study

**DOI:** 10.3389/frtra.2024.1370945

**Published:** 2024-04-04

**Authors:** Kaisa Ahopelto, Juulia Grasberger, Fernanda Ortiz, Agneta Ekstrand, Arno Nordin, Marko Lempinen, Ilkka Helanterä

**Affiliations:** ^1^ Department of Transplantation and Liver Surgery, University of Helsinki and Helsinki University Hospital, Helsinki, Finland; ^2^ Department of Nephrology, University of Helsinki and Helsinki University Hospital, Helsinki, Finland

**Keywords:** CMV, SPK, pancreas transplantation, kidney transplantation, valganciclovir

## Abstract

Cytomegalovirus (CMV) infections remain a common problem after solid-organ transplantation. We characterized the burden of CMV infections, and adverse events of CMV prophylaxis after simultaneous pancreas-kidney transplantation (SPK). We included all SPK patients (*n* = 236) since 2010 in our country. Immunosuppression was ATG, tacrolimus, mycophenolate, and steroids. Valganciclovir prophylaxis was given to all CMV D+/R− patients for six months, and to seropositive SPK patients for three months since February 2019. CMV DNAemia was monitored with quantitative PCR from plasma. Among D+/R− SPK recipients, post prophylaxis CMV infection was detected in 41/60 (68%) during follow-up. In seropositive SPK recipients with no prophylaxis, CMV infection was detected in 53/95 (56%), vs. 28/78 (36%) in those who received 3 months of prophylaxis (*P* = 0.01). CMV was symptomatic in 35 (15%) patients, of which 10 required hospitalization. Mean duration of viremia was 28 days (IQR 21–41). Leukopenia was detected in 63 (46%) of the 138 patients with valganciclovir prophylaxis. 7/122 (6%) of the CMV infections detected were defined as refractory to treatment, and three patients had confirmed ganciclovir resistance. SPK recipients experience a high burden of CMV infections despite CMV prophylaxis. Leukopenia is common during valganciclovir prophylaxis.

## Introduction

Without prophylaxis, approximately 10%–20% of patients will suffer from symptomatic CMV infection after kidney transplantation (1). The risk of CMV infections is highest among high-risk patients, i.e., CMV seronegative recipients of an organ from a seropositive donor (D+/R−), in whom CMV DNAemia can be detected in up to 40% of patients even after 6 months of antiviral prophylaxis with valganciclovir (2, 3). However, the highest burden of CMV disease for the health care system and individual patients is still among the CMV seropositive patients, even in countries with a lower prevalence of CMV seropositivity (4).

Patients with type I diabetes and diabetic nephropathy are potential candidates for simultaneous pancreas-kidney (SPK) transplantation, which is shown to be associated with potential benefits in survival and quality of life compared to kidney transplantation alone (5). With increased intensity of immunosuppression required for the transplanted pancreas, these patients may be at higher risk of infections, such as CMV.

Current guidelines recommend antiviral prophylaxis with valganciclovir for all patients after pancreas transplantation (except for D−/R− risk constellation) (6). However, in our clinical experience, the burden caused by CMV infections remain high despite prophylaxis. Furthermore, valganciclovir is not well tolerated, as leukopenia is a frequent complication among recipients of pancreas transplantation, who often receive lymphocyte-depleting induction therapy. In addition, although seldom, the lack of efficacy of valganciclovir for the treatment of CMV infection is seen in these patients. Relatively scarce recent literature has focused on infectious burden after SPK transplantation, and current occurrence and timing of CMV infections after pancreas transplantation are not accurately described.

The aim of this study was to characterize in detail the occurrence, timing, clinical burden of CMV infections, and adverse events of CMV prophylaxis after simultaneous pancreas-kidney transplantation.

## Materials and methods

### Patients

This is a retrospective study, using prospectively collected observational registry data, including all patients who have received a simultaneous pancreas-kidney transplantation between 2010 and July 2022 at Helsinki University Hospital. Data have been collected from the Finnish Transplant Registry, where the patients were identified.

The pancreas transplantation program was started at Helsinki University Hospital in 2010, and all patients with a minimum three months of follow-up until October 2022 were included in this study. Patients with SPK transplants are routinely followed up at the transplant center with annual or biannual visits in addition to more frequent follow up in the local central hospital.

### Immunosuppression and transplant procedure

The immunosuppressive protocol after SPK transplantation, and technical procedure of pancreas transplantation have been previously described in detail (7). Briefly, all patients received induction with a single-dose rabbit ATG (8 mg/kg), followed by tacrolimus-based triple-drug immunosuppression, with tacrolimus trough level target 10–12 µg/L during the first three months, mycophenolate dose 1,000 mg twice daily (or corresponding dose of mycophenolate sodium), and steroids with dose tapering and withdrawal usually during the first posttransplant year. Steroids remain a part of the immunosuppression in patients who experience rejection or have donor-specific antibodies at the time of transplantation. Pancreas was transplanted intra-abdominally with enteric proximal jejunal exocrine drainage.

### CMV prophylaxis and treatment of CMV infections

After SPK transplantation, CMV seropositive recipients transplanted before 2019 did not received routine CMV prophylaxis, similarly to the institutional policy in kidney transplantation, but were monitored for CMV DNAemia with two-week intervals for the first three months. Antiviral treatment was always initiated in case of symptoms and a positive DNAemia. In asymptomatic patients, treatment was initiated at the clinicians' discretion, but usually if the viral load exceeded 1,000 IU/ml. Since February 2019, CMV seropositive recipients of SPK transplants received valganciclovir prophylaxis for three months (dose 900 mg once daily, or adjusted for kidney function), followed by monitoring of CMV DNAemia with 3–4 weeks interval.

SPK recipients with D+/R− constellation received six months prophylaxis with valganciclovir (dose 900 mg once daily, or adjusted for kidney function), followed by monitoring of CMV DNAemia with 2–4 weeks interval. Frequent CMV monitoring continued for one year post transplantation.

CMV infections were treated primarily with valganciclovir (dose 900 mg twice daily or adjusted for kidney function). Intravenous ganciclovir was used in case of severe symptoms (such as severe gastrointestinal symptoms compromising drug absorption), or failure to respond to valganciclovir treatment. In case of refractory or resistant CMV infection, treatment was individually evaluated.

### Definitions

The following definitions were used which are consistent with the American Society of Transplantation Infectious Diseases Community of Practice and CMV Drug Development Forum recommendations for use in clinical trials (8, 9): Patients with a positive CMV-DNAemia were defined as suffering from CMV infection regardless of symptoms. CMV disease was defined as symptomatic CMV DNAemia accompanied by clinical signs or symptoms, which were retrospectively collected from the electronic medical records. CMV disease in the context of the current study is defined as “probable CMV disease”, as confirmation of tissue-invasive disease is not routinely done in our clinical practice. Recurrent CMV infection was defined as new CMV DNAemia after initially achieving clearance of viremia with at least a four-week period of undetectable DNAemia in between viremias during active surveillance. Refractory CMV infection was defined as described (10), i.e., failure to record at least 1 log10 decrease in viral load despite treatment with an adequate dose of antiviral medication for at least two weeks. Leukopenia was defined as total leukocyte count below 3.3 × 10^9^, the lower limit of the reference range in our laboratory system.

### Data collection

Clinical and laboratory data were collected from the Finnish Transplant Registry and hospital electronic medical records. The primary outcome variable was the occurrence of CMV infection and the occurrence of probable CMV disease. In addition, secondary outcome variables included hospitalization due to CMV infection, recurrence of CMV infection, occurrence of leukopenia leading to dose adjustments or medication discontinuation after transplantation, and detection of treatment failure with (val-)ganciclovir, or ganciclovir resistance.

### CMV diagnostics

CMV infections were diagnosed with quantitative detection of CMV DNAemia from plasma specimens, as described (3), and are reported as IU/ml. A conversion factor from cp/ml to IU/ml has been determined for the earlier quantitative PCR method (11), and was used in this study to convert the viral loads as comparable values. No major differences have occurred in diagnostic methods for CMV infection, nor in clinical practices regarding patient follow-up or CMV monitoring. Testing for genotypic CMV resistance was done only in case of clinical suspicion of resistance, and the genotypic resistance assay was performed at Sahlgrenska University Hospital, Gothenburg, Sweden.

### Statistical methods

Descriptive statistics were used as appropriate. Comparison between two groups were done with the Mann–Whitney *U*-test, or Fisher's exact test, as appropriate. Nonparametric statistics were chosen, as the distributions were not normal. Two-sided *P*-values <0.05 are considered significant. Calculations were performed with IBM SPSS (version 27, IBM Corporation, Somers, NY).

## Results

### Patients

Altogether 236 SPK patients were included. Baseline demographics are reported in Table 1. Altogether 60/236 (25%) SPK recipients were D+/R− and received 6 months of valganciclovir prophylaxis. In addition, 78/157 CMV seropositive recipients received three months valganciclovir. Nineteen patients were D−/R− and received no prophylaxis. Eight patients died (three of infections, three cardiopulmonary disease, and two malignancies) and 8 pancreas grafts were lost during follow-up, whereas only 2 patients returned to dialysis treatment after kidney graft loss during a median follow-up of 3.7 years.

**Table 1:** Demographic details of the patients who received a simultaneous pancreas-kidney transplantation between 2010 and July 2022.

Patient characteristics	SPK (*n* = 236)
Recipient age (years)	42 ± 8
Recipient male sex (%)	150/236 (64%)
Recipient BMI	24 ± 3
Donor age (years)	39 ± 14
Donor BMI	24 ± 3
Time in dialysis (months)	16 ± 12
Diabetes duration (years)	32 ± 9
HLA-AB-mismatch	2.7 ± 0.9
HLA-DR-mismatch	1.5 ± 0.6
CMV D+/R− (%)	60/236 (25%)
Induction immunosuppression	
ATG	236 (100%)
Basiliximab	0
Tacrolimus-based immunosuppression (vs. cyclosporine)	236 (100%)
Rejection treatment (%)	72/236 (31%)
Creatinine 1 year (mg/dl)	1.3 ± 0.8

### CMV infections

Detailed characteristics of CMV infections are presented in Table 2. CMV infection was detected in 122/236 (52%) SPK recipients. Among D+/R− SPK recipients, CMV infection was detected in 41/60 (68%), 9 of these were breakthrough infections during the valganciclovir prophylaxis. In seropositive recipients with no prophylaxis, CMV infection was detected in 53/79 (67%), vs. 28/78 (36%) in those who received 3 months of prophylaxis (*P* < 0.001). Asymptomatic infections were treated with valganciclovir until two weekly measurements of CMV plasma levels were normal, or at least two weeks.

**Table 2:** Comparison of the burden of CMV between different subgroups of recipients of simultaneous pancreas-kidney transplantation.

	Seropositive SPK recipients with prophylaxis (*N* = 78)	Seropositive SPK recipients without prophylaxis (*N* = 79)	D+/R− SPK recipients with prophylaxis (*N* = 60)
Leukopenia during valganciclovir prophylaxis (%)	31 (40%)	N/A	32 (53%)
Immunosuppressive dose reduction	8 (10%)		26 (43%)
G-CSF use	4 (5%)		12 (20%)
Valganciclovir discontinuation	11 (14%)		17 (28%)
CMV infection after transplantation (%)	28 (36%)*	53 (67%)	41 (68%)
Symptoms associated with CMV (%)	3/78 (4%)	12/79 (15%)	20 (33%)**
Leukopenia or thrombopenia	3/3 (100%)	1/12 (9%)	12/20 (60%)
GI symptoms	3/3 (100%)	9/12 (75%)	9/20 (45%)
Fever	0/3 (0%)	2/12 (17%)	10/20 (50%)
Malaise	0/3 (0%)	0/12 (0%)	5/20 (25%)
Timing of first CMV after transplantation (median and IQR)	193 (165–239)***	41 (31–61)	223 (162–264)
Duration of CMV DNAemia (days, median and IQR)	38 (28–57)****	26 (21–33)	32 (21–57)
Peak viral load (IU/ml, median and IQR)	235 (63–1,600)*****	1,302 (345–6,630)	3,215 (757–22,586)
Hospitalization due to CMV (%)	4/28 (14%)	1/53 (2%)	12 (29%)
Recurrence of CMV (%)	16/28 (57%)^#^	6/53 (11%)	17/41 (41%)
(Val-) ganciclovir treatment failure or ganciclovir resistance (%)	1/28 (4%)	1/53 (2%)	5/41 (12%)

* *P* = 0.01, compared to seropositive patients with no prophylaxis.

** *P* < 0.001, compared to seropositive patients.

*** *P* < 0.001, compared to seropositive patients with no prophylaxis.

**** *P* = 0.04, compared to seropositive patients with no prophylaxis.

***** *P* = 0.01, compared to seropositive patients with no prophylaxis.

^#^ *P* < 0.001, compared to seropositive patients with no prophylaxis.

All other differences are nonsignificant.

### Adverse events related to valganciclovir prophylaxis

Among the 138 SPK patients who received valganciclovir prophylaxis, leukopenia was detected in 63 (46%), G-CSF was used to treat neutropenia in 16 (12%), mycophenolate dose was adjusted in 34 (25%), and valganciclovir had to be discontinued prematurely in 28 (20%) patients (Table 2).

### Detailed description of CMV infections

CMV infection was symptomatic in 35 patients (15% of the 236 SPK patients), of which 10 required hospitalization and treatment with iv ganciclovir. No cases were life-threatening or required treatment in the intensive care unit. Median peak viral load was 1,600 IU/ml (IQR 291–9,900), and median duration of viremia 28 days (IQR 21–41). In symptomatic CMV infections, immunosuppression was adjusted, and mycophenolate dose reduced or discontinued.

Altogether 7/122 (6%) of the CMV infections detected in SPK patients were defined as refractory to treatment, and three patients had a genotypically confirmed ganciclovir resistance. Altogether 14 patients required treatment with iv ganciclovir, either due to failure of oral valganciclovir therapy (eight patients) or severe symptoms/high viral load at disease presentation (six patients).

Five patients had genotypic resistance tested, and three of these patients had a confirmed genotypic ganciclovir resistance. In one patient M4601 in UL97, and D413Y and G841A mutations in the UL54 gene were detected; in the second patient M460M/V and C603C/W mutations in UL97, and T503T mutation in UL54 gene were detected. In the third patient, H520P and C603W mutations were detected in the UL97 gene. Five patients were treated with letermovir (dose 480 mg once daily). Initial viral clearance was reported in all patients treated with letermovir. Longest duration of viremia was 334 days in a patient with confirmed genotypic resistance, despite treatment with letermovir. Recurrent CMV infections after primary clearance of viremia were detected in 39/122 (32%), and were asymptomatic.

## Discussion

In the current study we show that the burden of CMV infections remains very high among recipients of simultaneous pancreas-kidney transplants. CMV viremia was very frequent and was seen in 68% of D+/R− patients, a majority of these after six months valganciclovir prophylaxis, and in 36% of CMV seropositive patients who received three months of prophylaxis. CMV prophylaxis halved the rate of CMV viremias in seropositive patients in our study. Longer prophylaxis could be associated with a lower rate of post-prophylaxis infections, although no studies exist in prolonged prophylaxis after pancreas transplantation, and prophylaxis is associated with significant side-effects and costs. Valganciclovir prophylaxis was poorly tolerated, as leukopenia was detected in 40% of patients, and in 18% patients valganciclovir had to be prematurely discontinued due to leukopenia. Part of this is probably due to a cumulative effect of valganciclovir and immunosuppression.

Although CMV infections have been extensively studied after kidney transplantation, and among solid-organ transplant recipients in general, the existing literature of CMV infections after pancreas transplantation remains limited. According to a previous study including only 62 SPK patients, the incidence of CMV viremia was higher in SPK compared to kidney-only recipients despite valganciclovir prophylaxis of one to three months, and SPK recipients were more likely to develop a tissue-invasive CMV infection (12). In previous studies, the frequency of CMV infections among the CMV D+/R− SPK patients has been between 20%–60%, and similarly as after all solid organ transplants, CMV D+/R− patients are at highest risk of developing CMV viremia (12–14). A recent study described cumulative rates of clinically significant CMV infection to be 54.0% in D+/R− and 15.8% in R+ patient populations (15). The difference in prevalence compared to our study is likely due to us reporting CMV DNAemia and the other study reporting clinically significant CMV infections defined as either symptomatic disease or one needing antiviral treatment. Nevertheless, our findings confirm the high burden of CMV infections also after six months of prophylaxis among SPK patients.

In the current study, the frequency of CMV infections was high. While this probably relates to the high level of immunosuppression used among the SPK recipients (ATG induction, and tacrolimus-based regimen with higher mycophenolate does), it also highlights the challenge with simultaneous pancreas-kidney transplantation, in which high intensity of immunosuppression is needed in the background of multiple different tissue types (kidney, pancreas, duodenum), containing also more viral burden with reactivation capacity. Despite this, rejections in SPK patients occur relatively often with 18% of patients having either pancreas or kidney rejection post transplantation (16). SPK patients have more infections than kidney transplant recipients during the first year after transplantation (17). The most common infections were of gastrointestinal origin, especially *Chlostridioides difficile* and norovirus (17). Finding a balance between increased risk of infections and a tolerable rate of rejections is a challenge. In our own previous publications, the frequency of CMV infections has been approximately 20% after kidney transplantation (1), and the frequency of late-onset primary CMV infection after six months of prophylaxis has been approximately 40% (2, 3).

In the current guidelines (6), CMV prophylaxis is generally recommended after pancreas transplantation, and our current data support this recommendation. Among CMV seropositive patients with no prophylaxis, CMV infections occurred earlier, more frequently, and with a higher viral load compared to those who received prophylaxis. In addition, although duration of viremia was longer among patients who received prophylaxis, recurrent infections were less frequently seen. On the other hand, leukopenia was seen in 26% of the seropositive patients who received valganciclovir prophylaxis.

First reports on ganciclovir resistance were reported in the 1990s and were related to CMV retinitis treatment in AIDS patients, in whom the incidence of resistance increased over time with longer treatment (18). The frequency in solid organ transplant recipients is reported to be 2%–4% (19). Among 407 patients who received a pancreas transplant in a previous study (226 patients with SPK), ganciclovir resistance was detected in 4 patients (1%) (13). In our current study, ganciclovir resistance was confirmed in three patients, which is 1.3% of the total population, but 2.5% of the SPK patients treated with (val)ganciclovir for CMV infection. Of the CMV infections in our study, 6% were defined either refractory or resistant to ganciclovir treatment. Recently, alternative treatments for resistant/refractory CMV infections became available, most importantly maribavir (20). Also letermovir may offer a treatment option for SOT recipients with CMV infection and ganciclovir resistance (21). Five patients in our current cohort were successfully treated with letermovir for refractory/resistant CMV infection, before Maribavir was approved in Europe. No reliable estimates of the frequency of refractory CMV infections have been previously reported after solid organ transplantation. Persistent or refractory CMV infection are, however, common after hematopoetic cell transplantation (HCT) with frequencies reported even up to 30%–40% (22–24).

A fixed duration of three months of valganciclovir prophylaxis has been shown to reduce CMV disease but associated with neutropenia in nearly 40% of the recipients in a kidney transplant population (25). Valganciclovir prophylaxis causes higher rates of neutropenia compared to other viral prophylaxis in solid organ transplant patients even in the absence of induction therapy (26). A lower dose (450 mg daily) of valganciclovir was equally effective in preventing CMV disease than a higher dose (900 mg daily), but adverse effects like needing G-CSF or dose reduction of valganciclovir due to leukopenia were more common in the group receiving higher dose (27). Emerging of ganciclovir resistant strains may be a concern with the subtherapeutic valganciclovir dose used for prophylaxis and is currently not recommended (6).

Our study has some limitations of note, in addition to being a retrospective study. Although covering a national cohort of consecutive patients, all the patients were transplanted in a single center, and the results may not be generalizable to other SPK cohorts with possibly different immunosuppressive regimens. The high intensity of immunosuppression needed for pancreas transplantation, and thus high risk of adverse events should also be considered when informing potential candidates of the risks and benefits of pancreas transplantation, and our data may offer be useful information for clinical practice. On the other hand, the strength of our study is that it covers a relatively large cohort of consecutive SPK transplantations from the current era.

In conclusion, major disease burden is still associated with CMV infection after simultaneous pancreas-kidney transplantation. Although no severe or life-threatening infections were recorded, post prophylaxis infections were very frequent and valganciclovir prophylaxis was poorly tolerated.

## Data Availability

The original contributions presented in the study are included in the article/Supplementary Material. Requests to access the datasets should be directed to ilkka.helantera@hus.fi.
